# Relationships between feeding and glucose concentrations in healthy term infants during the first five days after birth—the Glucose in Well Babies Study (GLOW)

**DOI:** 10.3389/fped.2023.1147659

**Published:** 2023-03-24

**Authors:** Deborah L Harris, Philip J. Weston, Jane E Harding

**Affiliations:** ^1^ Newborn Intensive Care Unit, Waikato District Health Board, Hamilton, New Zealand; ^2^ School of Nursing, Midwifery & Health Practice, Faculty of Health, Te Herenga Waka, Victoria University of Wellington, Wellington, New Zealand; ^3^ Liggins Institute, University of Auckland, Auckland, New Zealand

**Keywords:** infant, newborn, feeding, glucose, continuous interstitial glucose monitoring

## Abstract

**Background:**

The World Health Organization recommends breastfeeding be commenced as soon as possible after birth. Amongst other benefits, early feeding is expected to support the metabolic transition after birth, but effects on blood glucose concentrations are controversial. We sought to describe the changes in interstitial glucose concentrations after feedings over the first five postnatal days.

**Participants and Methods:**

In healthy singleton term infants, all feeds were recorded using a smart phone app. Glucose concentrations were measured by blinded interstitial monitoring, calibrated by heel-prick capillary samples 2–4 times/d. Feeding sessions were included if a start and end time were recorded, and if the interval between the start of successive feeds was >90 min. The area under the glucose concentration curve (AUC) was calculated by trapezoidal addition from baseline (median of the 3 measurements before the beginning of the session). The maximum deviation (MD) was the greatest change in glucose concentration (positive or negative) from baseline to the next feeding session or 180 min, whichever came first. Data were analyzed using Stata V17 and are presented as mean (95% CI) in mmol/L.

**Results:**

Data were available for 62 infants and 1,770 feedings. The glucose response to breastfeeding was not different from zero on day 1 [day 1 AUC 0.05 (−0.00, 0.10), MD 0.06 (−0.05, 0.16)], but increased thereafter (day 3 (AUC 0.23 (0.18, 0.28), MD 0.41 (0.32, 0.50), day 5 AUC 0.11 (0.06, 0.16), MD 0.28 (0.18, 0.37), *p* < 0.001 for age effect). Glucose response increased with increased duration of breastfeeding (<30 min AUC 0.06 (0.02,0.09), MD 0.12 (0.04,0.19), >30 min AUC 0.20 (0.16, 0.23) MD 0.37 (0.30, 0.44), *p* < 0.001 for duration effect) and this was observed even in the first 2 days (<30 min AUC-0.02 (−0.06, 0.03), MD −0.06 (−0.15, 0.03), >30 min AUC 0.12 (0.08, 0.16), MD 0.19 (0.11, 0.27), overall *p* < 0.001 for age x duration interaction). In feeding sessions that were not breastfeeding, the glucose response was greater after formula than after expressed human milk [AUC 0.29 (0.15, 0.29), MD 0.48 (−0.12, 0.61)], and greater after feed volumes >20 ml than <10 ml [20–30 ml AUC 0.19 (0.01, 0.27), MD 0.23 (−0.01, 0.46)].

**Conclusion:**

The glucose response to feeding in the days after birth increases with postnatal age and duration of the feeding episode. Breastfeeding for <30 min has little effect on glucose concentrations in the first two days.

## Introduction

Current best practice recommendation is to establish breastfeeding as soon as possible after birth (1). Among the many benefits of breastfeeding, establishing early feeds is thought to support the neonatal metabolic transition (2). Infants at risk of hypoglycemia are particularly encouraged to feed in order to reduce the likelihood of hypoglycemic episodes, and infants found to be hypoglycemic are likewise encouraged to feed along with other treatments (3–6). Yet, in the first 48 h after birth when hypoglycemia is most common, it has been reported that formula feeding, but neither breastfeeding nor giving expressed human milk to hypoglycemic infants increased blood glucose concentration (7).

Two other reports have also shown that feeding healthy infants in the first hours after birth is not associated with an increase in blood glucose concentrations, regardless of milk type (8, 9). However, there are few data about the changes in glucose concentrations after feeding in the first few days after birth in healthy infants, or the factors that may influence this such as sex, postnatal age, gestational age, feeding type, and feeding duration.

Current data are also limited by intermittent glucose measurements, which cannot be used to determine details of the time course and magnitude of changes in glucose concentrations after a feed. Continuous glucose monitoring provides a glucose measurement approximately every 5 min and is safe and accurate in newborns (10).

Therefore, the aim of this prospective observational study was to determine the feeding-related changes in interstitial glucose concentrations in a cohort of healthy infants over the first 5 days after birth, and the factors that may influence these changes.

## Materials and methods

### Study population

The GLOW study was a prospective observational cohort study and has been previously reported (11–13). Eligible infants were healthy singleton term infants born in Hamilton, New Zealand, between November 2015 and August 2017. Each baby underwent capillary heel-prick sampling for measuring glucose concentrations over the first 5 days (4 on the 1st day, and 2 on each subsequent day), as well as continuous interstitial glucose monitoring from as soon after birth as possible. Infants were fed according to maternal choice, and parents recorded all feeds using a commonly used breastfeeding application (Feed Baby Pro, Penguin Apps, Version 21.0.5, Victoria, Australia). Mothers did not undertake antenatal breastmilk expression, and no donor milk was used. The researchers and families were blinded to all glucose concentrations and remained so until the data collection phase was complete and statistical analysis plans were finalized.

Glucose concentrations were measured on either an Epoc® blood analyzer (Siemens Healthineers, Erlangen, Germany), or blood gas analyzer if the baby was still in the hospital (Radiometer ABL800 FLEX, Copenhagen, Denmark). Both systems use glucose oxidase methods. Interstitial glucose data (continuous glucose monitor, CGM) were obtained using Ipro2 subcutaneous sensors (Medtronic Minimed Northridge, CA, USA) and were recalibrated according to a previously published algorithm (14). The interstitial glucose output gave a mean concentration every 5 min.

### Data management

Feeding data were downloaded from the app when the baby had completed the study. Each breastfeed was recorded as a left or right breast with the start and end time, while formula feeds were recorded as volumes with start and end times. Breastfeeds with a recorded duration ≤1 min were excluded.

A feeding session was separated from the previous and subsequent feed by >30 min and could be made up of one or more feed events (13). Breast feeding could be unpaired (one feed from one breast only), paired (first on one side and then on the other), or cluster (three or more feeds). Feeding sessions could also be from a bottle using either formula or expressed human milk (EHM). The milk given in each feeding session was classified as breast, formula, or mixed.

### Statistical analysis

A feeding session had associated interstitial glucose data if the CGM data were available from the beginning of the feeding session up to the beginning of the next feeding session or for 180 min, whichever came first, with no data dropouts of ≥3 consecutive data points (15 min). Feeding sessions were excluded from this analysis if the time to the next feeding session was <90 min, if the age was >120 h, or if the duration was not recorded. The baseline interstitial glucose concentration was the median of the 3 measurements before the beginning of the feeding session. The area under the glucose concentration curve (AUC) was calculated by trapezoidal addition from the baseline and expressed in mmol/L. The maximum deviation (MD) was the greatest change in glucose concentration from the baseline (positive or negative) from the beginning of the feeding session to the beginning of the next feeding session or 180 min, whichever came first. Mean change in interstitial glucose concentrations from baseline was calculated at 5-minute intervals.

The changes in interstitial glucose concentrations during and following feeding sessions were classified as “rising” if the interstitial glucose concentration showed a positive deviation from baseline throughout the first 90 min, with highest deviation >0.3 mmol/L; “falling” if the interstitial glucose concentration showed a negative deviation throughout the first 90 min, with lowest deviation of <−0.3 mmol/L; “flat” if the deviations remained between −0.3 and +0.3 mmol/L; or “wandering” for all other patterns.

Mixed model regression was used to determine the predictors of AUC and MD accounting for repeated measures within infants. Confidence intervals of the mean change in glucose concentration were obtained using mixed model regression adjusted for the non-independence of repeated feedings sessions within the same baby and the previous interstitial glucose concentration. The growth *z-*scores were calculated using the WHO standards (15) Analyses were performed using Stata Version 17.

## Results

Sixty-seven infants completed the GLOW study, and 62 (93%) were included in this analysis. Their mean (SD) birthweight was 3,566 (342) grams and gestation 40.0 (1.2) weeks (Table 1). These 62 infants had 1,770 feeding sessions with matched interstitial signal for a mean duration of 106 (28) hours in the first 120 h after birth. Of the feeding sessions 1,654 (93%) were solely breastfeeding. For the remaining 116 feeding sessions, 38 (33%) were solely formula, 35 (30%) were solely EHM and the remaining 43 (37%) were mixed.

**Table 1:** Description of the infants, feeding sessions, and interstitial glucose concentrations.

Infants	*n* = 62
Birthweight (grams)	3,566 (342)
Birthweight z score	0.3 (0.7)
Gestation (weeks)	40.0 (1.2)
Male	40 (65)
Vaginal Delivery	52 (84)
Feeding Sessions	*n* = 1770
Duration of feeding (minutes)	44 (36)
Time to next feed (minutes)	210 (78)
Feeding sessions
Breast feeding	1,654 (93)
Unpaired	687 (39)
Paired	706 (40)
Cluster	261 (15)
Formula alone	38 (2)
Formula with breastfeeding	20 (1)
EHM alone	35 (2)
EHM with breastfeeding	18 (1)
EHM with formula	5 (0)
Milk type
Human	1,707 (96)
Formula	38 (2)
Mixed	25 (1)

Data are mean (SD) or number (percent). EHM means expressed human milk.

**Figure 1:**
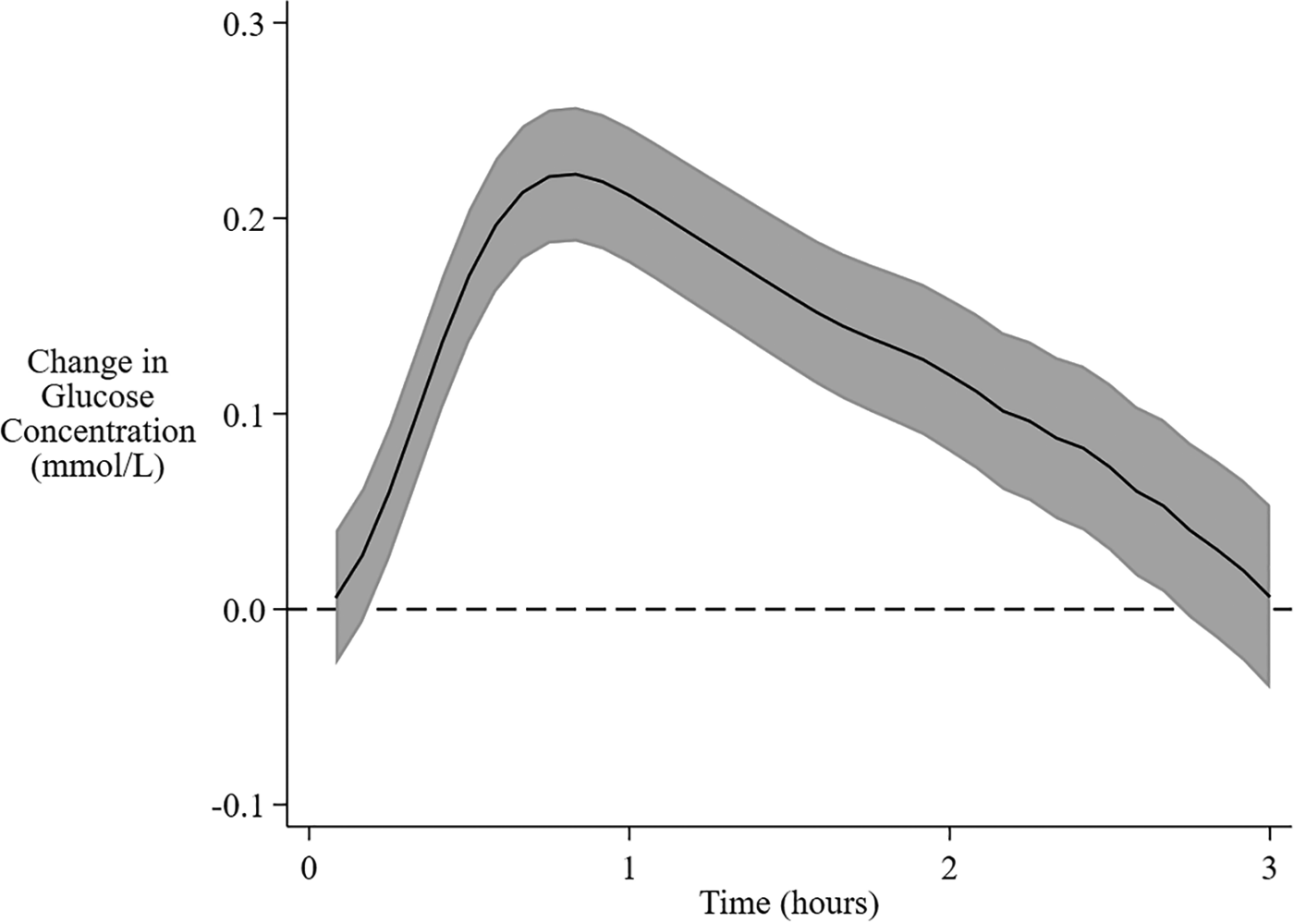
Overall mean change in interstitial glucose concentration from baseline following feeding. The solid central line is the overall mean change in interstitial glucose concentration from baseline over 3 h after the beginning of a feeding session. The shaded area encompasses the 95% confidence limits.

There was substantial variability in the interstitial glucose response to feeding, with the AUC ranging from −1.98 to 1.84 mmol/L, and the MD from −3.50 to 3.10 mmol/L. On average, interstitial glucose concentrations increased after the beginning of a feeding session, peaking at 50 min, and then declined back to baseline by 3 h (Figure 1).

**Figure 2:**
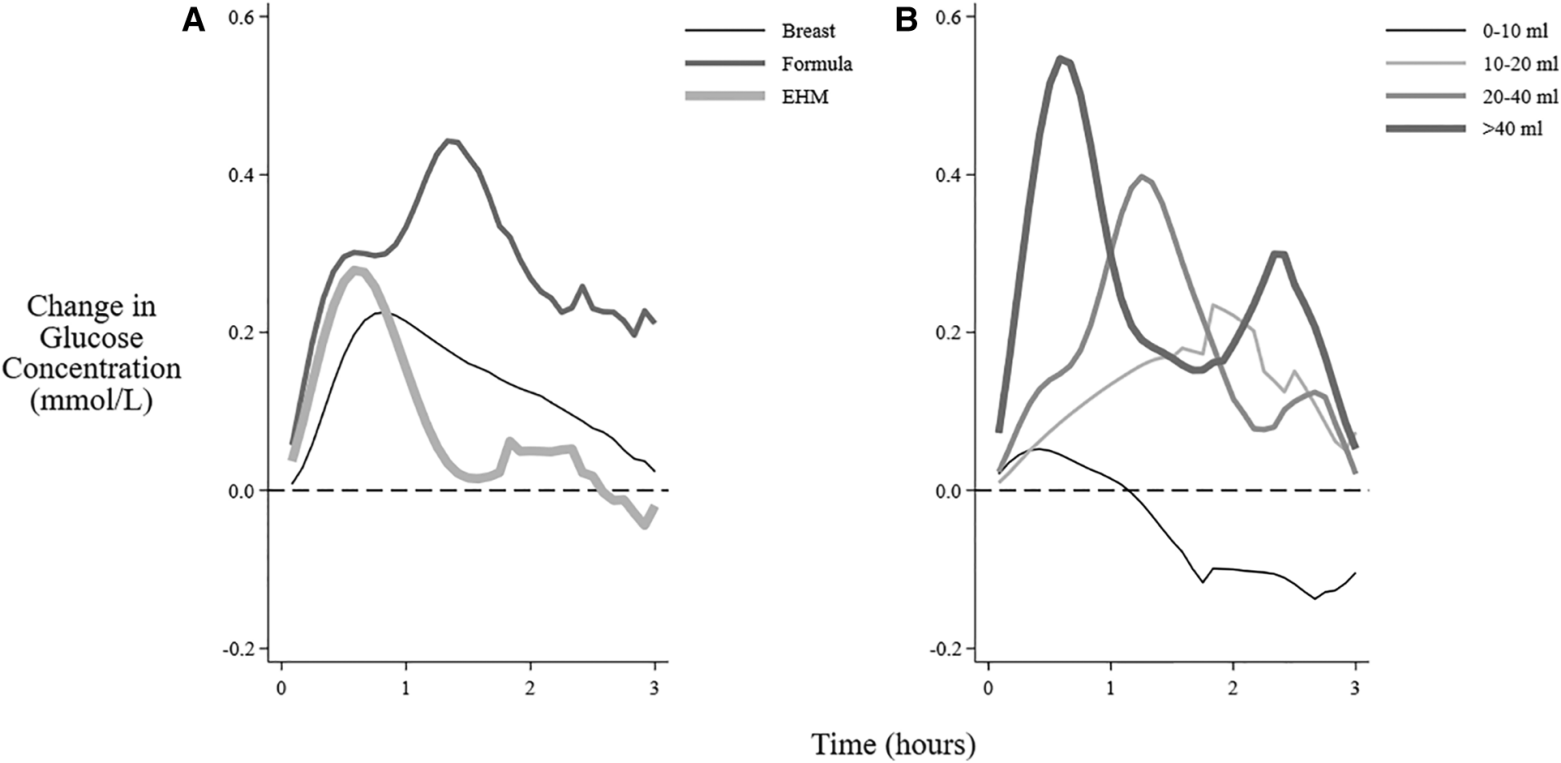
Mean change in interstitial glucose concentrations after feeding different milk types and volumes. The mean change in interstitial glucose concentration from baseline over 3 h after the beginning of different feeding sessions: (**A**) Feeding types (**B**) Volume of feeding (feedings that also included breastfeeding excluded). EHM, expressed human milk.

Formula feeding sessions were associated with a higher increase in mean interstitial glucose concentration and higher AUC but not MD compared with EHM feeding sessions (Figure 2A, Table 2). Greater milk volume (>20 ml) was also associated with a higher increase in mean concentration and higher AUC and MD (Table 2, Figure 2B). There were insufficient formula feeding sessions on day 1 (*n* = 3) or day 2 (*n* = 10) to draw any conclusions about age-related changes after formula milk feeding.

**Table 2:** Change in interstitial glucose concentration after feedings sessions that were not breastfeeding.

			Area under the Curve			Maximum Deviation		
			mmol/L			mmol/L		
		*n*	Estimated change	95% CI	*p*	Estimated change	95% CI	*p*
Milk	EHM	35	(0.08)	ref		(0.23)	ref	
	Formula	38	0.29	0.15, 0.29	<0.001	0.48	−0.12, 0.61	0.19
Feeding volume							
	<10 ml	18	(0.05)	ref		(0.04)	ref	
	10–20 ml	13	0.17	−0.04, 0.29	0.14	0.18	−0.10, 0.46	0.22
	20–30 ml	15	0.19	0.01, 0.27	0.03	0.23	0.01, 0.46	0.04
	30–40 ml	7	0.3	0.04, 0.47	0.02	0.65	0.41, 0.90	<0.001
	>40 ml	20	0.29	0.03, 0.46	0.03	0.71	0.21, 1.20	0.005

CI means confidence intervals. EHM means expressed human milk. Estimated change = mixed model regression estimation of change from reference category (ref) which is presented in brackets as the change from baseline. For example EHM was associated with a mean area under the curve of 0.08 mmol/L, and formula with an additional 0.29 mmol/L.

**Figure 3:**
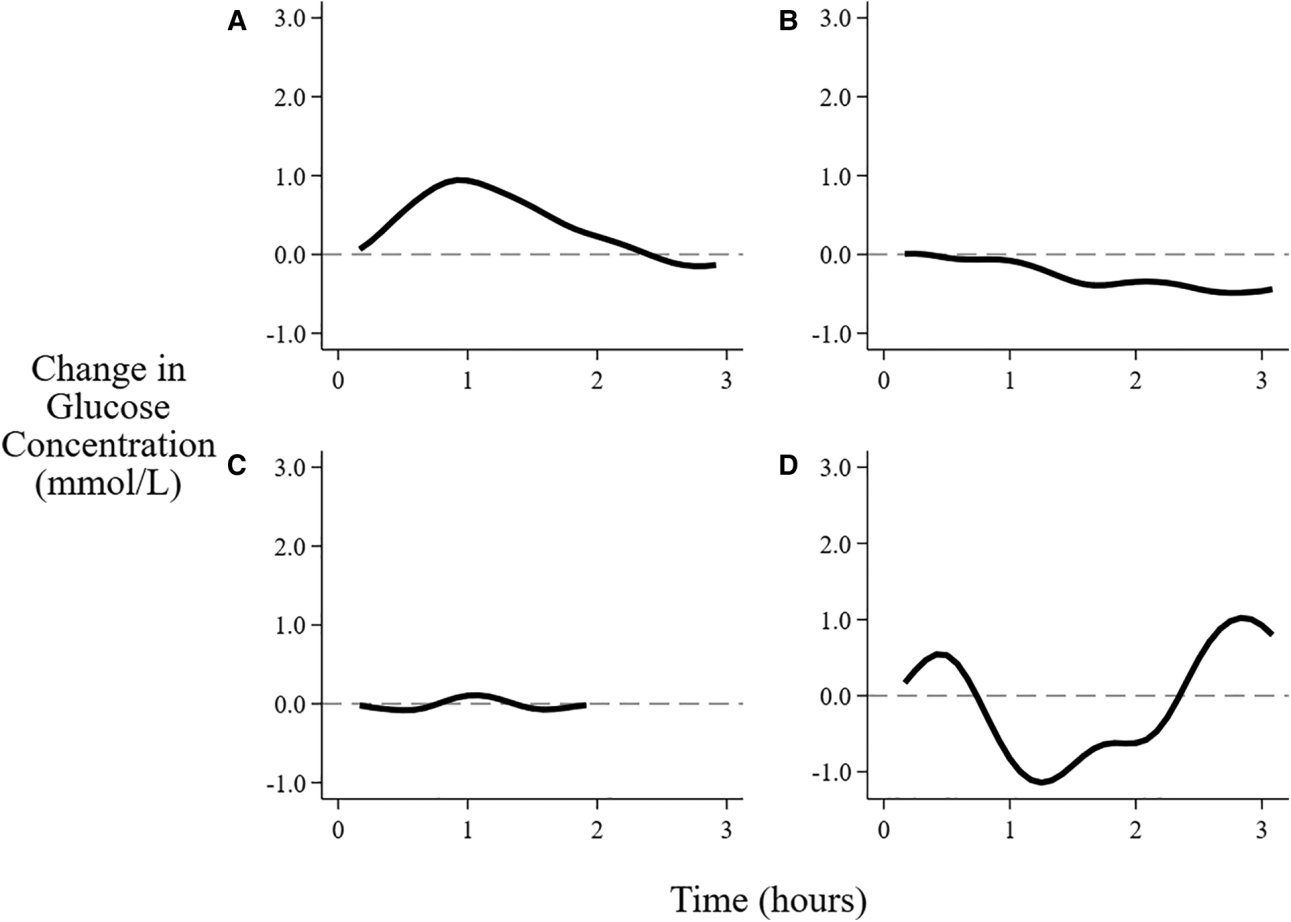
Patterns of changes in interstitial glucose concentration after feeding. (**A**) Rising, (**B**) Falling, (**C**) Flat, (**D**) Wandering. See text for definitions of patterns.

For solely breastfeeding sessions, the patterns of interstitial glucose concentrations were rising in 44% (721/1,654) of all sessions (Figure 3), with the proportion increasing from 26% (165/630) in the first two days to 55% (364/662) on days 4–5 (Table 3). The flat pattern was most common in the first two days (48%, 302/630) and unusual after day 3 (4%, 28/662). However, both the decreasing (between 9% and 21%) and wandering (between 13% and 24%) patterns were consistently seen on all the postnatal days.

**Table 3:** Patterns of changes in interstitial glucose concentrations associated with breastfeeding sessions over the first 5 days.

Day	Rising		Falling		Flat		Wandering		Total
1	74	(26.8)	32	(11.6)	122	(44.2)	48	(17.4)	276
2	91	(25.7)	33	(9.3)	180	(50.8)	50	(14.1)	354
3	192	(53.0)	49	(13.5)	73	(20.2)	48	(13.3)	362
4	204	(57.6)	55	(15.5)	17	(4.8)	78	(22.0)	354
5	160	(51.9)	63	(20.5)	11	(3.6)	74	(24.0)	308
Total	721	(43.6)	232	(14.0)	403	(24.4)	298	(18.0)	1,654

Data are the number (%) of feeding sessions on each day.

**Figure 4:**
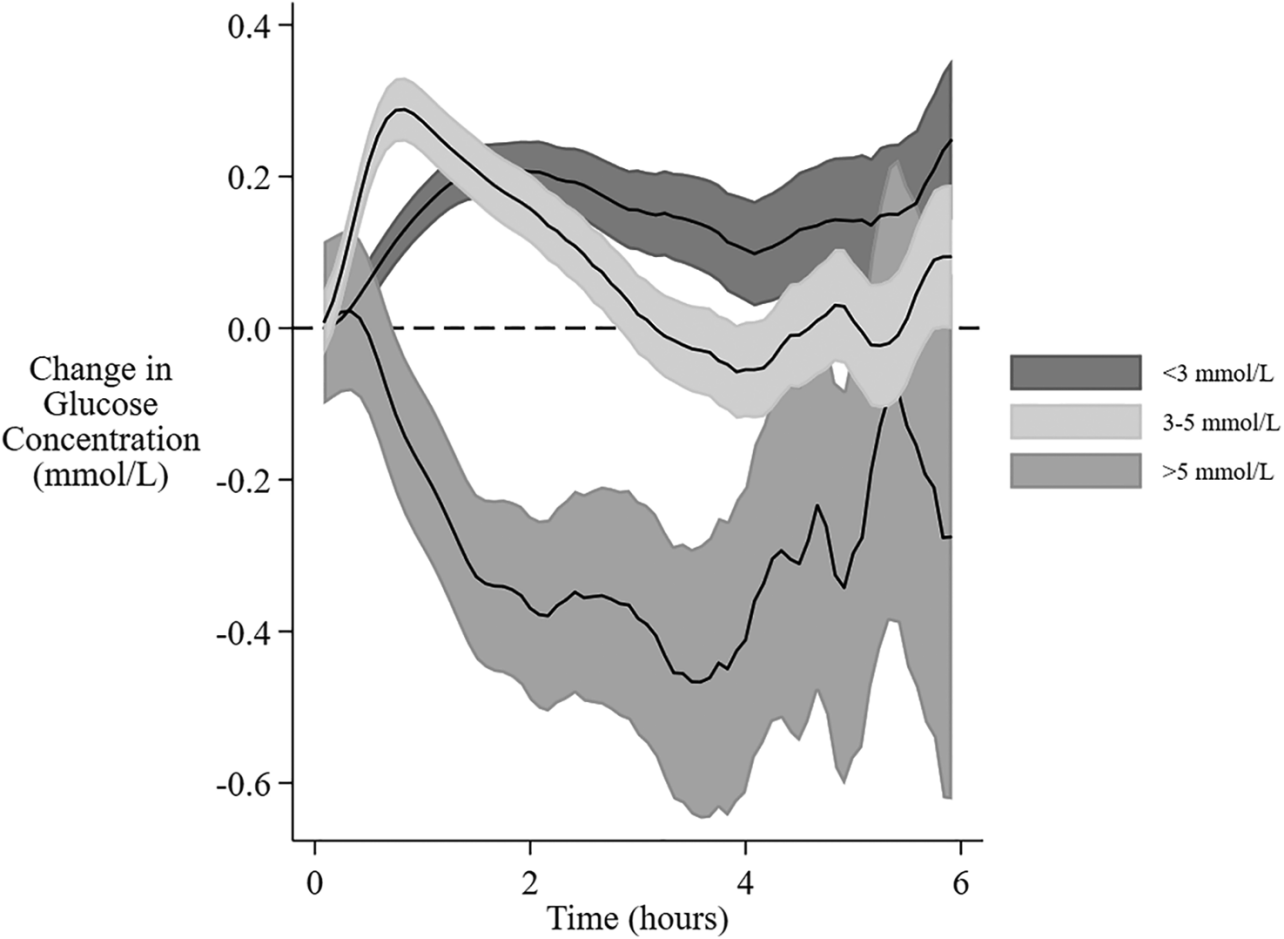
Change in interstitial glucose concentration after feeding at different baseline glucose concentrations. The mean change and 95% confidence intervals for interstitial glucose concentrations from baseline over 6 h from the beginning of a feeding session with different baseline glucose concentrations. The gradual expansion in confidence limits is due to decreasing amounts of data as the time between feeding sessions increases.

**Figure 5:**
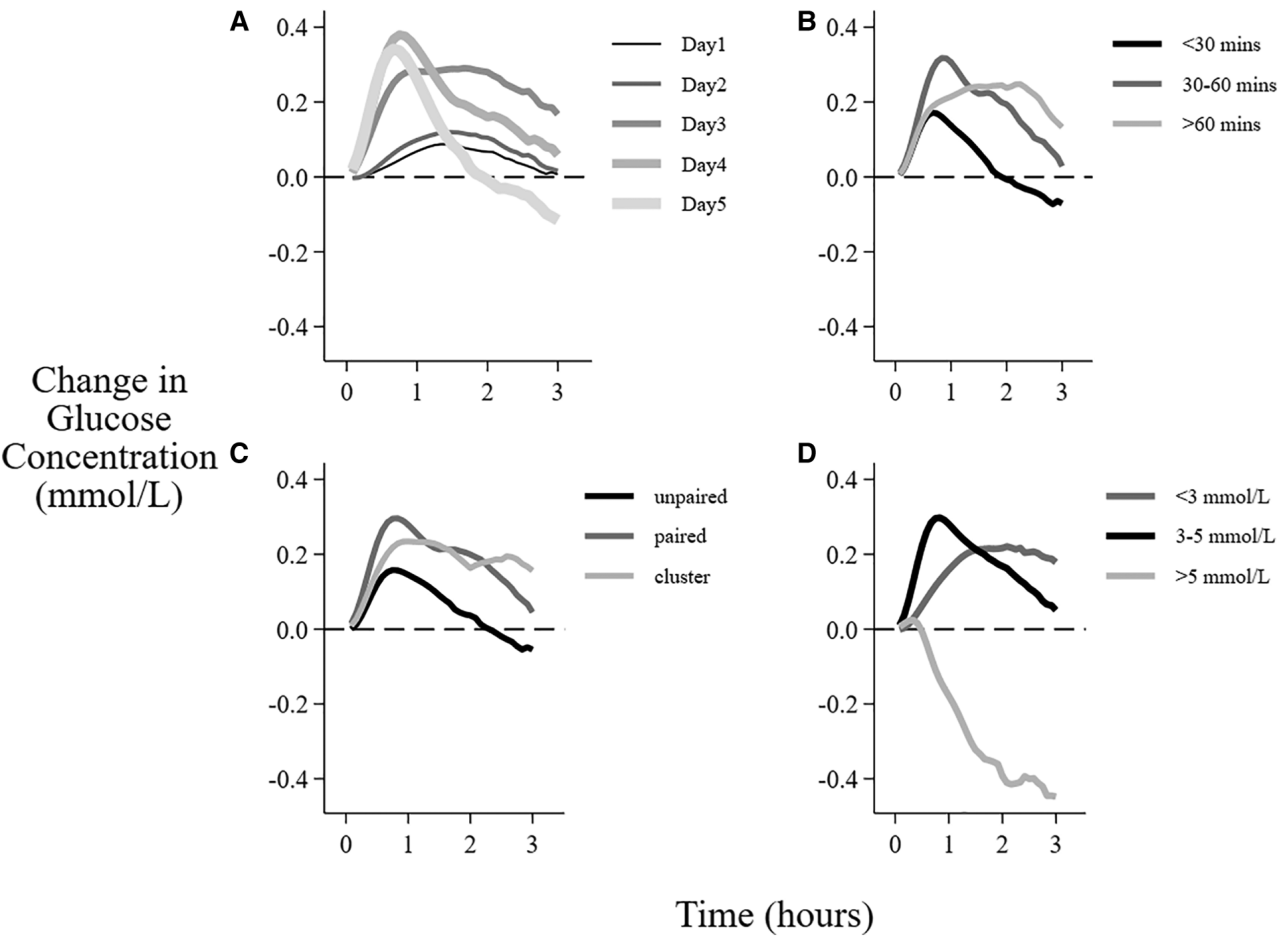
Factors associated with the mean change in interstitial glucose concentration after breastfeeding sessions. Data are the mean change in glucose concentration from baseline over 3 h after the beginning of feeding sessions with different (**A**) postnatal age, (**B**) duration of the feeding, (**C**) feeding type, (**D**) baseline glucose concentration.

Of the 1,770 feeding sessions available, the interval between feedings was prolonged beyond 4 h for 659, and was 4 to 5 h for 313, >5 to 6 h for 201, and >6 h for 145. The mean interstitial glucose was not different from baseline after 3 h. However, if the baseline was low (<3 mmol/L) the mean glucose concentration remained above baseline throughout the 6 h, whereas if the baseline was high (>5 mmol/L) the mean glucose concentration remained below baseline for up to 5 h. (Figure 4). A baseline glucose concentration >5 mmol/L was associated with a negative AUC and MD (Figure 5D, Table 4).

**Table 4:** Change in interstitial glucose concentration after breastfeeding sessions of different types in infants with different baseline glucose concentrations, sex, gestation and birthweight.

			Area under the Curve			Maximum Deviation		
			mmol/L			mmol/L		
		*n*	Estimated change from ref	95% CI	*p*	Estimated change from ref	95% CI	*p*
Breastfeeding type	Unpaired	687	(0.06)	ref		(0.12)	ref
	Paired	706	0.13	0.08, 0.17	<0.001	0.25	0.16, 0.34	<0.001
	Cluster	261	0.13	0.07, 0.19	<0.001	0.22	0.10, 0.34	0.001
Baseline interstitial glucose concentration	<3 mmol/L	299	(0.16)	ref		(0.26)	ref
	3–5 mmol/L	1,203	0.02	−0.03, 0.07	0.53	0.10	−0.00, 0.21	0.06
	>5 mmol/L	152	-0.40	−0.48, −0.32	<0.001	−0.73	−0.89, −0.56	<0.001
Sex	Female	560	(0.14)	ref		(0.29)	ref
	Male	1,094	−0.01	−0.07, 0.05	0.83	−0.04	−0.15, 0.08	0.55
Gestation	37–39 weeks	798	(0.17)	ref		(0.34)	ref
	40–42 weeks	856	−0.06	−0.11, 0.00	0.05	−0.15	−0.25, −0.04	0.005
Birthweight Z-score	<0	516	(0.14)	ref		(0.23)	ref
	≥0	1138	0.00	−0.06, 0.07	0.89	0.05	−0.07, 0.17	0.40

CI means confidence intervals, Estimated change means mixed model regression estimation of change from reference category (ref), which is presented in brackets as the change from baseline. Models are adjusted for repeated measures within each baby.

There was no clear interstitial glucose response to feeding on day 1. However, the response to feeding increased and peaked on days 3–4 (Figure 5A, Table 5). There was no change in mean glucose response to feeding in the first 2 days when the breastfeeding duration was <30 min, whereas longer feeds were followed by increased interstitial glucose concentrations on all days (Table 5).

**Table 5:** Change in interstitial glucose concentration after breastfeeding sessions of different durations on different postnatal days.

			Area under the Curve			Maximum Deviation		
			mmol/L			mmol/L		
		*n*	estimated change	95% CI	*p*	estimated change	95% CI	*p*
Overall Breastfeedings	1,654	0.14	0.11, 0.17	<0.001	0.27	0.21, 0.32	<0.001
Age	Day1	276	0.05	−0.00, 0.10	0.06	0.06	−0.05, 0.16	0.03
	Day 2	354	0.07	0.03, 0.12	0.002	0.1	0.01, 0.20	0.03
	Day 3	362	0.23	0.18, 0.28	<0.001	0.41	0.32, 0.50	<0.001
	Day 4	354	0.21	0.16, 0.25	<0.001	0.44	0.35, 0.53	<0.001
	Day 5	308	0.11	0.06, 0.16	<0.001	0.28	0.18, 0.37	0.001
Duration	<30 m	669	0.05	0.01, 0.09	0.008	0.11	0.03, 0.19	0.004
	30–60 m	586	0.19	0.15, 0.23	<0.001	0.36	0.28, 0.44	<0.001
	>60 m	399	0.21	0.16, 0.25	<0.001	0.38	0.28, 0.47	<0.001
Multiple Model
Age < 3d, duration <30m	271	−0.02	−0.06, 0.03	0.41	−0.06	−0.15, 0.03	0.19
Age < 3d, duration >30m	359	0.12	0.08, 0.16	<0.001	0.19	0.11, 0.27	<0.001
Age ≥ 3d, duration <30m	428	0.11	0.07, 0.15	<0.001	0.23	0.15, 0.31	<0.001
Age ≥ 3d, duration >30m	596	0.25	0.21, 0.28	<0.001	0.48	0.41, 0.55	<0.001

CI means confidence intervals. Estimated change means mixed model regression estimation of change in interstitial glucose concentration from baseline, adjusted for repeated measures within each baby.

There was a smaller change in glucose concentration after unpaired breastfeeding compared with paired and cluster breastfeeding (Figure 5C), with mean difference in AUC of 0.13 mmol/L, CI 0.07, 0.19 (Table 4). Although unpaired breastfeedings were generally shorter in duration than other breastfeedings, this difference between the change in glucose concentration after unpaired vs. paired and cluster feeding remained after adjusting for feed duration, (adjusted mean difference in AUC 0.06 mmol/L, CI 0.00, 0.11, *p* = 0.04).

The volume of milk given was lower on days 1–2 than on days 3–5 for both formula (mean (SE): 13.2 (6.7) vs. 32.0 (5.6) ml, *p* = 0.005) and EHM (6.9 (6.8) vs. 36.1 (6.4) ml, *p* < 0.001).

Infants with lower gestation (37–39 weeks) had a higher glucose response to feeding, but sex and z-score were not related to the change in glucose concentration after feeding (Table 4).

## Discussion

We describe the relationship between feeding and the change in interstitial glucose concentrations in a cohort of healthy term appropriately grown infants over the first 5 days. Our data show overall the mean interstitial glucose concentration increased following the start of a feeding session, returning to baseline after approximately 3 h. However, there was considerable variability in the changes in glucose concentrations related to the postnatal and gestational age of the baby, the type and duration of breastfeed, feed volume, and the baseline interstitial glucose concentration. Factors associated with an increase in glucose concentration were postnatal age >48 h, breastfeeding for >30 min and feeding from both breasts.

Most infants in our study were exclusively breastfed, and mothers were supported to establish breastfeeding soon after birth. Overall, our data show healthy newborns have a pattern of change in interstitial glucose concentrations related to postnatal age. On day 1, there was no significant change in interstitial glucose concentrations following feeding, and the most common pattern of change was flat. However, after 48 h there is a more predictable increase in interstitial glucose concentrations following feeding, and the most common pattern was rising. It remains unclear why the glucose response in the first 48 h is less than in subsequent days. Possible contributors include downregulation of plasma insulin concentrations following birth (16), the changing composition of the mother's milk over the first 48 h (17), and suckling-induced release of vagal mediated hormones including gastrin and cholecystokinin (18). Further, following the first 48 h the mother experiences lactogenesis II which is associated with an increasing volume of breast milk, and milk intake increases considerably (19). Coupled with this change in available volume, both the frequency and duration of breastfeeding sessions increase (13). These findings together suggest the supply of milk may be a key influence on the observed changes in the interstitial glucose concentration response to feeding.

Our findings show the duration of breastfeeding was also associated with the change in interstitial glucose concentrations, and this was detected as early as day 1, with feeding sessions >30 min associated with a greater glucose response. It is possible that a longer feeding session may increase the transfer of milk to the infant, or conversely, that greater available milk volume may encourage the infant to feed for longer, in either case thereby provide more glucose precursors. It is also possible that the longer duration of suckling mediates a more sustained hormone release. Feeding from both breasts (paired or cluster feeding) was also associated with an increase in interstitial glucose concentrations independent of the duration of feeding, although the reason for this is unclear. Since feeding is the most common initial treatment for transitional neonatal hypoglycemia, further investigation may be warranted to determine whether longer breastfeeding sessions and feeding from both breasts should be recommended for infants at risk of hypoglycemia.

Early changes in blood glucose concentrations have been difficult to measure in healthy infants, and hence there are few reports. However, consistent with our findings, in a retrospective analysis from Arkansas, blood glucose concentrations were similar in infants whose blood glucose concentrations were measured before or after the first feed [before first feed, mean (SD) 51.8 (11.9) mg/dl vs. after first feed 55.5 (13.3) mg/dl] (9). The authors also found the initial blood glucose concentrations were similar regardless of whether the infants were breastfed, formula fed or combination fed. Another study measured blood glucose concentrations in 75 healthy infants at 1 h after birth and found that glucose concentrations were similar in infants who were breastfed (*n* = 22), formula fed (*n* = 24) or not fed (*n* = 29) (8). Together, these reports are consistent with our findings suggesting that the timing of the initial feed has little influence on early changes in blood glucose concentrations. However, we were unable to further explore the effect of type of milk in our cohort because few infants received formula.

We found the mean change in glucose response varied related to the baseline blood glucose concentration. Lower baseline glucose concentrations (<3 mmol/L) were associated with a sustained blood glucose increase, which did not return to baseline even after 6 h. Conversely a higher baseline glucose concentration (>5 mmol/L) was associated with a decreasing mean blood glucose concentration and was sustained for up to 5 h. These findings appear to suggest that homeostatic mechanisms regulating glucose concentrations are functioning over timeframes of several hours even in the first few days after birth.

We are not aware of previous reports on the effect of feeding on interstitial glucose concentrations in healthy infants. The strengths of our study are that participants were all term healthy newborns, who were cared for using practices recommended for most infants born in high income settings. Feeding data were captured prospectively by the parents who were motivated research partners. All infants wore a continuous glucose monitor and the method of glucose analysis for the heel prick blood tests used glucose oxidase, which enabled accurate calibration with the continuous monitor, to support the reliability of our findings. However, the continuous glucose monitor requires an hour for calibration following insertion. Therefore, we were unable to evaluate the first feeding session for many infants. Further, there are a limited number of formula feedings, which limited our ability to make comparisons between breastfeeding sessions and infant formula feeding. In addition, families who participated in this study are not representative of the overall population which limits the generalizability of our findings.

In summary, healthy term infants demonstrate considerable variability in the interstitial glucose response to feeding. On average, interstitial glucose concentrations peak at 50 min, and then decline back to baseline by 3 h after feeding. Although breastfeeding is the best way to feed newborns (2), our findings show breastfeeding for <30 min has little effect on glucose concentrations in the first two days. However, breastfeeding for >30 min, feeding from both breasts, greater feed volume, postnatal age >48 h and early term birth were associated with greater changes in in glucose concentrations after feeding.

## Data Availability

The original contributions presented in the study are included in the article/supplementary material, further inquiries can be directed to the corresponding author/.
